# Surgical Treatment of Pulmonary Artery Angiosarcoma - A Ten-Year Experience

**DOI:** 10.21470/1678-9741-2023-0441

**Published:** 2025-06-11

**Authors:** Alexander Edemskiy, Oksana Vasiltseva, Elena Kliver, Natalya Novikova, Dmitry Sirota, Alexander Chernyavskiy

**Affiliations:** 1 Department of Aorta and Coronary Artery Surgery, E. Meshalkin National Medical Research Center, Ministry of Health of the Russian Federation, Novosibirsk, Russian Federation

**Keywords:** Pulmonary Artery, Pulmonary Hypertension, Endarterectomy, Pneumonectomy, Vascular Resistence

## Abstract

**Introduction:**

Pulmonary artery angiosarcoma is a rare and extremely severe tumor. Our study summarizes the clinical data of patients treated for pulmonary artery angiosarcoma over the period of 2010-2020.

**Methods:**

We retrospectively analyzed cases of surgical treatment of patients with diagnosis of pulmonary artery angiosarcoma at our center. Data of operative findings, short-term follow-up, and the long-term results were reviewed where available.

**Results:**

The 30-day mortality rate was six (67%) out of nine patients. Three (33%) patients were discharged. Data on pulmonary vascular resistance in the earlyand long-term postoperative periods were assessed if possible. Certain computed tomography signs have been identified that can be used to suspect pulmonary artery angiosarcoma and make a differential diagnosis with chronic thromboembolic pulmonary hypertension.

**Conclusion:**

The surgical treatment of choice is pneumonectomy with contralateral pulmonary endarterectomy. Oncological vigilance regarding angiosarcoma in occlusive-stenotic lesions of the pulmonary artery is extremely important. Patients’ assessment must be carried out in an expert cardiothoracic surgery center with the involvement of an oncological crew.

## INTRODUCTION

**Table:** 

Abbreviations, Acronyms & Symbols	
CT	= Computed tomography
CTEPH	= Chronic thromboembolic pulmonary hypertension
MRI	= Magnetic resonance imaging
PAS	= Pulmonary artery angiosarcoma
PE	= Pulmonary embolism
PET	= Positron emission tomography
PTE	= Pulmonary thromboendarterectomy
PVR	= Pulmonary vascular resistance
RHC	= Right heart catheterization
SVC	= superior vena cava
WHO	= World Health Organization

Pulmonary artery angiosarcoma (PAS) is an extremely rare disease. It was first described in 1923 by Mandelstamm^[1]^. From the point of view of cardiology and cardiovascular surgery, patients with PAS come to the attention usually with a diagnosis of pulmonary embolism (PE) or chronic thromboembolic pulmonary hypertension (CTEPH). Approximately 300 cases have been described^[2]^, mostly as case reports. Considering the rather limited number of cases, there are few small case series describing the management of these tumors^[2]^. The largest series have been described by pathologists^[3]^. PAS usually originates from the intimal cells and is referred to as intimal sarcoma. It represents only 2% of soft tissue sarcomas^[4]^. Early diagnosis is always difficult. A diagnosis of angiosarcoma depends on immunohistochemical findings^[5]^, and effective treatments for this tumor have not yet been established^[6]^. PAS has a poor prognosis with most patients dying within a few months after their first clinical manifestations^[7^,^8]^.

## METHODS

We retrospectively reviewed medical records and follow-up clinical data (where applicable) of all patients with diagnosis of PAS. Between January 2010 and December 2020, a total of nine patients were operated on due to PAS at our center. During the period from January 2021 to the present day, there were no cases of PAS in our clinic. The medical data of all patients were reviewed to evaluate the clinical characteristics, operative findings, short-term follow-up, and the long-term results where applicable. Patients were contacted by telephone interview or direct telemedicine consultation if possible.

The clinical manifestation in seven (78%) patients corresponded to CTEPH. Of the patients who complained about progressive dyspnea, three (33%) were in World Health Organization (WHO) functional class II and six (67%) were in WHO functional class III. In one patient, a prolonged fever of unknown origin was observed as the onset of the disease. Among other complaints it should be noted chest pain in five (56%) patients, syncope in three (33%) patients, and hemoptysis in one (11%) patient.

Usual preoperative work-up consists of clinical evaluation, chest X-ray, echocardiography, ventilation-perfusion scintigraphy, computed tomography (CT), and right heart catheterization (RHC). Chest X-ray usually showed signs of pulmonary hypertension (n = 9) and suspected metastatic lung disease (n = 3). According to echocardiography, there were also no specific PAS data: signs of pulmonary hypertension (n = 9), right ventricular systolic dysfunction (n = 7), and tricuspid insufficiency of varying degrees (n = 9). It should be noted that in four cases, echocardiography specialists described thrombotic masses in one of the main pulmonary arteries without indicating that these masses are atypical. Ventilation-perfusion scanning (n = 9) showed in all cases a mismatch between normal or subnormal ventilation and a strongly altered perfusion. Chest CT demonstrated typically the presence of a large quantity of endoluminal material proximally in the main pulmonary artery. The usual description of CT scans by radiologists was consistent with CTEPH (n = 8). Extravascular spreading of the lesion was mentioned in two cases. Magnetic resonance imaging (MRI) was of some help in reassessment the endovascular component of the “thrombi” in three patients after CT study. Pulmonary angiograms demonstrated usual CTEPH signs with proximal (level I or II according to University of California San Diego CTEPH classification)^[7]^ pulmonary artery lesions (n = 9). Despite the fact that pulmonary angiography is the “gold standard” for surgeons in the diagnosis of group IV pulmonary hypertension, there are no clear specific signs of PAS. Two patients with an initial PAS diagnosis underwent positron emission tomography (PET). Tumor biopsy in these patients was performed during RHC. Moreover, these biopsies were performed in referring oncological clinics after telemedicine consultation with our center. In our series, according to the preoperative examination, there was never any preoperative data on the involvement of the pulmonary valve in the pathological process and/or its dysfunction.

In two (23%) patients, the diagnosis of PAS was established preoperatively using PET. In the case of an initial PAS diagnosis, the original plan for surgery consisted of pneumonectomy ipsilateral to the tumor and contralateral thromboendarterectomy. In cases of initial CTEPH diagnosis, all patients underwent isolated bilateral pulmonary thromboendarterectomy (PTE). In seven (77%) patients, PTE was performed together with tumor removal (chronic thrombi + tumor); in two (23%) cases, PTE was supplemented with ipsilateral pneumonectomy. In all cases, pneumonectomy was performed after removing the aortic cross-clamp during rewarming period. All patients with PAS underwent bilateral thromboendarterectomy, except for cases with pneumonectomy, where contralateral thromboendarterectomy was also performed. All cases of PAS were unilateral. In six (67%) cases, PAS originated from the left lower lobe branch of the pulmonary artery, and in three (33%) cases, it was from the right lower lobe branch. The technical details of PTE are similar to the surgery for CTEPH^[8]^. PTE is performed during circulatory arrest under deep hypothermia (18°C), with the intention of removing neoplastic obstructive material from each pulmonary artery and its lobar or segmental branches. As mentioned above, seven patients were operated on with an initial CTEPH diagnosis. At the beginning of the thromboendarterectomy stage, it was the atypical nature of the thrombus that was the reason for raising the question of immediate changing the intraoperative diagnosis. In all cases, immediate intraoperative biopsy was performed. Also in all cases, after urgent cytological verification of the PAS diagnosis, the removal of tumor masses from the lumen of the pulmonary artery was continued. A distinctive feature of this procedure is the fragile nature of the tumor masses with difficulty in finding the true dissection plane. Typically, in one of the lobar arteries it is not possible to radically perform thromboendarterectomy, and this is the source of the tumor process. This is the basis for performing ipsilateral pneumonectomy. As in CTEPH, patients with PAS develop significant systemic collateral blood flow from bronchial arteries. In this regard, it is impossible to perform lobectomy due to the extremely high risk of bleeding under conditions of heparinization and deep hypothermia. Pneumonectomy was performed on cardiopulmonary bypass during patients rewarming by experienced thoracic surgeon. These procedures were performed via median sternotomy. Ipsilateral main pulmonary artery was reconstructed under bypass using xenopericardium. Histological examination was performed in all cases.

## RESULTS

The mean cardiopulmonary bypass and aortic cross-clamping times were 213 (121; 421) and 91 (79; 131) minutes, respectively. The mean deep hypothermic circulatory arrest time was 38 (33;47) minutes. The dynamics of pulmonary vascular resistance (PVR) are presented in Table 1 (where applicable).

**Table 1:** Pulmonary vascular resistance (PVR) and mortality rate before and after surgery.

	PVR before surgery, dyn·s/cm^-5^	PVR after surgery^#^, dyn·s/cm^-5^	Mortality
Patient 1	988	559	Yes
Patient 2	973	678	Yes
Patient 3	1107	743	Yes
Patient 4	1221	Not applicable	Yes
Patient 5	904	568	Yes
Patient 6	1103	Not applicable	Yes
Patient 7	993	187	No
Patient 8	1021	271	No
Patient 9	878	371	No

# Measurements were collected the next day after surgery

The 30-day mortality rate was six (67%) cases. Mortality was registered in patients who were operated on with an initial diagnosis of СTEPH. Causes of death included multiple organ failure in four (44%) patients and failure to wean from bypass due to severe reperfusion pulmonary edema and high-grade pulmonary hypertension in two (22%) cases. Extracorporeal membrane oxygenation was not connected because the prognosis in patients with intraoperatively verified angiosarcoma is extremely poor. Intraoperative or early postoperative RHC (if applicable) showed relatively high PVR and residual pulmonary hypertension along with reperfusion edema in lethal cases (Table 1).

Three (33%) patients were discharged with further recommendation of treatment and observation by an oncologist. The three surviving patients were operated on last in chronological order (2019-2020). The follow-up period after surgery in one patient was > 12 months, in two patients it was up to 12 months. The life expectancy of one patient was 2.5 years; he died from the progression of distant spinal metastases. The other two patients were followed up for 12 months and did not experience a recurrence of the disease, but contact with them was subsequently lost.

**Fig 1:**
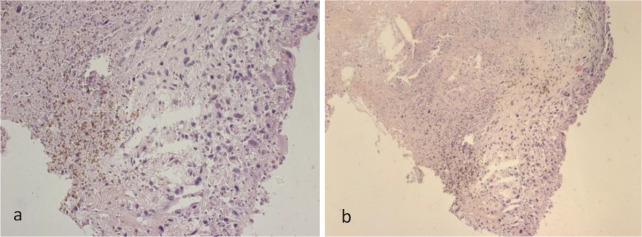
Pulmonary angiosarcoma. Samples for histological examination were obtained by intraoperative biopsy. Cells of various shapes and sizes with polymorphic nuclei with the presence of pathological mitoses and eosinophilic cytoplasm. a) ×200; b) ×100. Staining with hematoxylin and eosin.

Among the survivors were those who also underwent pneumonectomy. According to the control CT-angiography of the pulmonary artery in surviving patients, no recurrence of the disease was detected. According to pathomorphological examination, high-grade sarcoma was revealed in all cases (Figure 1).

## DISCUSSION

The symptoms of PAS are non-specific that makes an early diagnosis rather challenging. PAS usually mimics CTEPH or acute pulmonary thromboembolism, because the clinical manifestations of PAS are remarkably similar to them. If we look at the classification of pulmonary hypertension, the group IV, in addition to CTEPH, includes neoplastic obstructive pulmonary artery lesion and angiosarcoma. Indeed, in our series, the initial diagnosis in most of the patients was CTEPH. As mentioned by S. Mussot et al.^[9]^, when carrying out the differential diagnosis of CTEPH and PAS, we should also remember about such pathological conditions as pulmonary arteritis (Behçet disease, Takayasu disease), primary lung cancer, mediastinal tumors, tumor emboli into the pulmonary artery (from extrathoracic cancers), hydatic emboli, and fibrosing mediastinitis. Therefore, it is important to assess the patient by a multidisciplinary team in the expert pulmonary hypertension center with the possible use of telemedicine technologies.

**Fig. 2:**
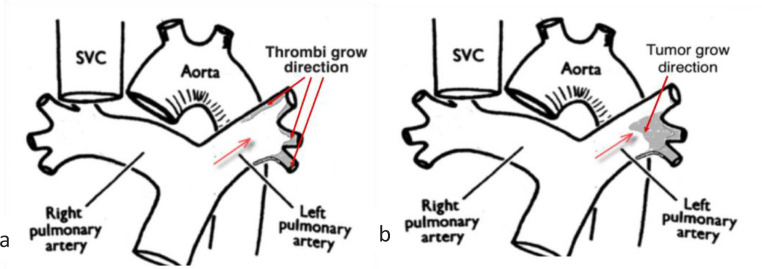
Difference between chronic thromboembolic pulmonary hypertension (a) and pulmonary artery angiosarcoma (b) lesion according to computed tomography. Red arrow corresponds to blood flow in pulmonary artery and directions of thrombi and tumor grow. SVC=superior vena cava.

Unfortunately, CT does not allow for an accurate PAS diagnosis. In our opinion, the only potential typical sign of PAS on CT scans may be the character of PAS growth of to the intravascular lumen. Usually, thrombotic lesion due to CTEPH represents on CT as narrowing or occlusion of pulmonary artery branches along the blood flow (Figure 2). Unlike CTEPH, in PAS tumor originates from the distal pulmonary arteries and grows proximally.

This appearance may help in suspicion of PAS and perform additional assessment (PET, biopsy) during preoperative differential diagnosis.

In our series of patients, according to preoperative evaluation, there was no involvement of the pulmonary valve in the pathological process. Although, according to S. Mussot et al.^[9]^, pulmonary valve may be involved in pathological process. Bleisch et al.^[10]^ reported that in 60 patients with PAS, the main pulmonary artery was involved in 100% of cases, the pulmonary valve in 57%, the right ventricle in 25%, the right pulmonary artery in 67%, and the left pulmonary artery in 60%. As mentioned by M. Correale et al.^[11]^, the involvement of the right ventricular outflow tract and pulmonary valve by extraluminal infiltration and small or no change in size after appropriate anticoagulation at the ultrasound follow-up are in favor of diagnoses other than PE.

F-18 fluorodeoxyglucose PET/CT is a tool which made it possible to suspect a tumor lesion and send the patient for a biopsy during RHC and thereby perform a differential diagnosis. Gadolinium-enhanced MRI may help differentiate between thrombotic lesions and vasculature tumors^[12]^.

A feature of our series is that according to RHC, the mean preoperative PVR was 1021 dyn·s/cm-5, which suggests that patients come to our center with high degree of pulmonary hypertension and PAS progression. However, in a much larger series of patients with PAS described by S. Mussot et al.^[9]^, preoperatively degree of pulmonary hypertension was half as much as in our series. Only preoperative endovascular biopsy during RHC in two patients allowed us to diagnose PAS and plan treatment strategy. Preoperative biopsy was performed at the referring oncology clinic after telemedicine consultation with our center. There were not any procedural complications during preoperative biopsy.

When analyzing the surgical treatment of PAS in the historical aspect, pneumonectomy was the first performed option since the 1960’s^[12]^. With the further development of cardiothoracic surgery, PTE, both isolated and in conjunction with resection procedures, was also proposed. In our series, the combination of PTE (contralaterally) and ipsilateral pneumonectomy, despite the extremely large surgical aggression, made it possible to radically perform the intervention and avoid serious adverse events in short term follow-up. Nowadays, in our opinion, among the surgical treatments of PAS, this strategy is preferable, while an accurate preoperative diagnosis is important. When analyzing mortality in our series, the main reasons are early residual pulmonary hypertension and reperfusion edema, which are the basis for the development of further multiple organ failure in the early postoperative period. Isolated PTE does not provide radical PVR reduction due to tumor obstruction of pulmonary artery branches. At the same time, unlike CTEPH, the search for a layer for endarterectomy in certain situations with PAS is practically impossible due to the tumor invasion of the artery wall. According to our 10-year experience, the mortality rate is quite high - six out of nine patients. Moreover, mortality took place in patients with an initial diagnosis of CTEPH and chronologically at an earlier period. This mortality level is consistent with those of other authors^[9]^, considering the extremely low number of case series descriptions in this area of cardiothoracic surgery.

All discharged patients were referred to experienced oncologists for decision about chemotherapy. Multimodality therapy, such as the combination of radiotherapy and immunotherapy (recombinant interleukin-2) and that of surgery and chemotherapy, has also proven to be effective^[13]^.

This series highlights the need to manage patients with suspect pulmonary arterial hypertension or CTEPH in adequate referral centers with specific and multi-professional expertise (heart and thoracic imaging). We should remember risk factors for PAS: radiotherapy for breast cancer, Stewart-Treves syndrome, infection such as filariasis, toxins including arsenic, anabolic steroids, vinyl chloride, and thorium dioxide, neurofibromatosis type 1, Maffucci syndrome, Klippel-Trénaunay syndrome, and some genetic mutations (*e.g.*, BRCA1 and BRCA2)^[14]^.

Thus, oncological alertness in the presence of an atypical clinical presentation of the disease, especially if CTEPH is suspected, the pattern of thrombus growth according to imaging modalities, as well as the use of MRI, PET, and biopsy during RHC will help in the diagnosis of PAS. In this case, together with oncothoracic surgeons, it is possible to discuss radical surgical treatment, as well as chemotherapy and/or radiotherapy as part of a combined treatment strategy. Today, this approach can lead to favorable short-term and long-term results.

### Limitations

Our study had some limitations. First, a rather small number of patients were reported. Second, this was a retrospective study, and the data were collected from a single center. At the same time, this can be explained by a rather rare pathology.

## CONCLUSION

The diagnosis of PAS is rather challenging. These tumors are rare and mimic pulmonary emboli, which are much more common. Making a conclusion despite the high perioperative mortality, with the accumulation of experience of the multidisciplinary team of the CTEPH center, it is possible to avoid mortality and extend the life of patients with this disease.
